# The Story of a Living Temple

**Published:** 1903-04

**Authors:** 


The Story of a Living Temple. A Study of the Human Body. By Frederick M. Rossiter, B. S., M. D., and Mary Henry Rossiter, A. M. Duodecimo, pp. 348. Chicago: Fleming H. Revell Co. 1902.

The authors present a physiology book for young readers, written in an attractive semi-allegorical style. It ought to succeed in its object, which is to instil in the young mind that respect and admiration for the body which leads to due regard for the laws of hygiene. The book is attractively printed and bound. M. J. F.

